# Stakeholder engagement in European brain research: Experiences of the Lifebrain consortium

**DOI:** 10.1111/hex.13747

**Published:** 2023-03-29

**Authors:** Isabelle Budin‐Ljøsne, Barbara B. Friedman, William F. C. Baaré, David Bartrés‐Faz, Rebecca B. Carver, Christian A. Drevon, Klaus P. Ebmeier, Anders M. Fjell, Paolo Ghisletta, Richard N. Henson, Rogier Kievit, Kathrine S. Madsen, Laura Nawijn, Sana Suri, Cristina Solé‐Padullés, Kristine B. Walhovd, Enikő Zsoldos

**Affiliations:** ^1^ Department of Food Safety Norwegian Institute of Public Health Oslo Norway; ^2^ Department of Psychology, Center for Lifespan Changes in Brain and Cognition University of Oslo Oslo Norway; ^3^ Danish Research Centre for Magnetic Resonance, Centre for Functional and Diagnostic Imaging and Research, Copenhagen University Hospital—Amager and Hvidovre Copenhagen Denmark; ^4^ Department of Medicine, Faculty of Medicine and Health Sciences, Institute of Neurosciences University of Barcelona Barcelona Spain; ^5^ Department of Communications Norwegian Institute of Public Health Oslo Norway; ^6^ Vitas AS Oslo Norway; ^7^ Department of Nutrition, Institute of Basic Medical Sciences, Faculty of Medicine University of Oslo Oslo Norway; ^8^ Department of Psychiatry, Oxford Centre for Human Brain Activity, Wellcome Centre for Integrative Neuroimaging, Warneford Hospital University of Oxford Oxford UK; ^9^ Methodology and Data Analysis Group, Faculty of Psychology and Educational Sciences University of Geneva Geneva Switzerland; ^10^ Faculty Council of the Faculty of Psychology UniDistance Suisse Brig Switzerland; ^11^ Swiss National Centre of Competence in Research LIVES University of Geneva Geneva Switzerland; ^12^ MRC Cognition and Brain Sciences Unit University of Cambridge Cambridge UK; ^13^ Cognitive Neuroscience Department Donders Institute for Brain, Cognition and Behavior, Radboud University Medical Center Nijmegen The Netherlands; ^14^ Radiography, Department of Technology University College Copenhagen Copenhagen Denmark; ^15^ Department of Psychiatry, Amsterdam Neuroscience, Amsterdam UMC Vrije Universiteit Amsterdam Amsterdam The Netherlands; ^16^ Department of Psychiatry, Amsterdam Neuroscience, Amsterdam UMC University of Amsterdam Amsterdam The Netherlands

**Keywords:** brain health, brain research, Lifebrain, stakeholder engagement

## Abstract

**Introduction:**

Stakeholder engagement remains scarce in basic brain research. However, it can greatly improve the relevance of investigations and accelerate the translation of study findings to policy. The Lifebrain consortium investigated risk and protective factors influencing brain health using cognition, lifestyle and imaging data from European cohorts. Stakeholder activities of Lifebrain—organized in a separate work package—included organizing stakeholder events, investigating public perceptions of brain health and dissemination. Here, we describe the experiences of researchers and stakeholders regarding stakeholder engagement in the Lifebrain project.

**Methods:**

Stakeholder engagement in Lifebrain was evaluated through surveys among researchers and stakeholders and stakeholders' feedback at stakeholder events through evaluation forms. Survey data were analysed using a simple content analysis approach, and results from evaluation forms were summarized after reviewing the frequency of responses.

**Results:**

Consortium researchers and stakeholders experienced the engagement activities as meaningful and relevant. Researchers highlighted that it made the research and research processes more visible and contributed to new networks, optimized data collection on brain health perceptions and the production of papers and provided insights into stakeholder views. Stakeholders found research activities conducted in the stakeholder engagement work package to be within their field of interest and research results relevant to their work. Researchers identified barriers to stakeholder engagement, including lack of time, difficulties in identifying relevant stakeholders, and challenges in communicating complex scientific issues in lay language and maintaining relationships with stakeholders over time. Stakeholders identified barriers such as lack of budget, limited resources in their organization, time constraints and insufficient communication between researchers and stakeholders.

**Conclusion:**

Stakeholder engagement in basic brain research can greatly benefit researchers and stakeholders alike. Its success is conditional on dedicated human and financial resources, clear communication, transparent mutual expectations and clear roles and responsibilities.

**Public Contribution:**

Patient organizations, research networks, policymakers and members of the general public were involved in engagement and research activities throughout the project duration.

## 1 INTRODUCTION

Research funders increasingly require health projects to engage stakeholders in all phases of research processes and governance to inform decision‐making processes,^1^, ^2^, ^3^ promote mutual learning and understanding between researchers and stakeholders^4^, ^5^ and facilitate the rapid translation of scientific findings into practical policy, including prevention as well as clinical practice.^6^ Stakeholders are ‘individuals, organizations or communities directly interested in the processes and outcomes of a project, research or policy endeavour’.^7^ In health research, stakeholders usually include healthcare professionals, policymakers, industry representatives, patients and their caregivers and advocates, the research participants and the general public.^8^ Stakeholder engagement refers to the process of involving stakeholders in research activities in which they are interested or by which they are affected to support shared understanding and effective decision‐making.^7^ Stakeholder engagement can take different forms but often includes activities allowing stakeholders to influence the conception, development and dissemination of research projects and facilitate the exchange of views and experiences.^8^

Stakeholder engagement in clinical brain research is common and encouraged in contexts where, for instance, the views of patients are needed to test the acceptance or efficacy of a treatment to inform clinical practice,^9^, ^10^, ^11^ or to understand their experiences of disease progression.^10^, ^12^ In stark contrast, stakeholder engagement remains scarce in projects conducting basic science research on the structure and function of the brain.^13^ This may partially be due to the nature of the research that, although providing important new and translatable knowledge, often produces findings of limited immediate clinical implications and may thus be perceived by researchers to be less relevant for stakeholders.^13^ However, there are a few notable exceptions. The Human Brain Project^14^ develops a research infrastructure for investigating brain biology and regularly interacts with stakeholders through the organization of stakeholder boards and citizen meetings to discuss the ethical, social and philosophical implications of neurological research.^15^ The Australian Brain Initiative, an alliance of public and private actors in brain sciences,^16^ engages researchers, healthcare professionals, policymakers, industry representatives and patient advocates in the development of national guidelines for ethically and socially robust neuroinnovation.^16^

Principles and guidelines for Patient and Public Involvement have been developed, thereby providing researchers with concrete guidance on engaging different groups with research into neurodegenerative and brain diseases.^17^, ^18^ The guidelines suggest avenues for identifying relevant individuals and stakeholder organizations to engage in research, define their roles in the different phases of research projects and establish working agreements. However, knowledge about how to engage stakeholders in basic brain research in a way that is meaningful and does not put unreasonable demands on the parties involved remains limited. A stakeholder engagement process is highly context‐dependent and demands resources in terms of logistical effort, time and funding.^19^ It might also prove difficult to maintain stakeholders' interest over time and keep the initial momentum going,^20^ thus leading projects to limit their stakeholder engagement activities to the first stages of research.^1^ Adequate stakeholder engagement should cover all phases of the research process. Learning from the experiences of other basic science projects with stakeholder engagement may be a useful guide to meaningful engagement in future projects.

This paper explores the experiences of researchers and stakeholders involved in a large collaborative brain research project regarding stakeholder engagement. It reports on results from evaluation surveys conducted among researchers and stakeholders and written feedback, elaborates on challenges and opportunities encountered when engaging stakeholders and provides recommendations for stakeholder engagement in future basic research projects. We hope that the recommendations will be relevant for future large‐scale health research projects, also outside the field of brain research.

### 1.1 The Lifebrain consortium

Lifebrain was founded in January 2017 by the European Union's Horizon 2020 programme to conduct basic brain research exploring environmental, social, occupational and lifestyle factors affecting brain development, cognitive function and mental health at different stages of life.^21^ The consortium investigated neuroscientific data, including brain imaging, demographic, cognitive, lifestyle, physical and mental health, blood markers and genetic data, from approximately 5200 participants across 14 project sites in Europe.^21^ The consortium was organized into seven work packages responsible for different components of the project, including a work package dedicated to stakeholder engagement. This work package was allocated 5.8% of the consortium's total salary budget (580,000 EUR) and had approximately 35,000 EUR for organizing stakeholder activities during the 5.5 years of the project. Researchers in the consortium's partner institutions were invited to contribute to the work package voluntarily, and an interdisciplinary work package team led by a researcher with expertise in research ethics and stakeholder engagement was established across countries. Work package members brought expertise within environmental sciences, psychology, neuropsychology, neuroscience, psychiatry, neurology, blood biomarkers and communication.

### 1.2 Stakeholder engagement in Lifebrain: Objectives and activities

Initially, the consortium aimed to exchange views with stakeholders and thereby benefit from their experiences and perspectives about how to promote brain health, increase public trust in brain research and enable the rapid uptake of research results in healthcare and public health policies. The consortium envisioned building a network of stakeholder organizations interested in brain health, cognition and mental health located in the countries of the project partners (Norway, Sweden, Germany, United Kingdom, Spain, Denmark, The Netherlands and Switzerland) or acting at European level, with whom a relationship was either already established or desirable.

**Figure 1:**
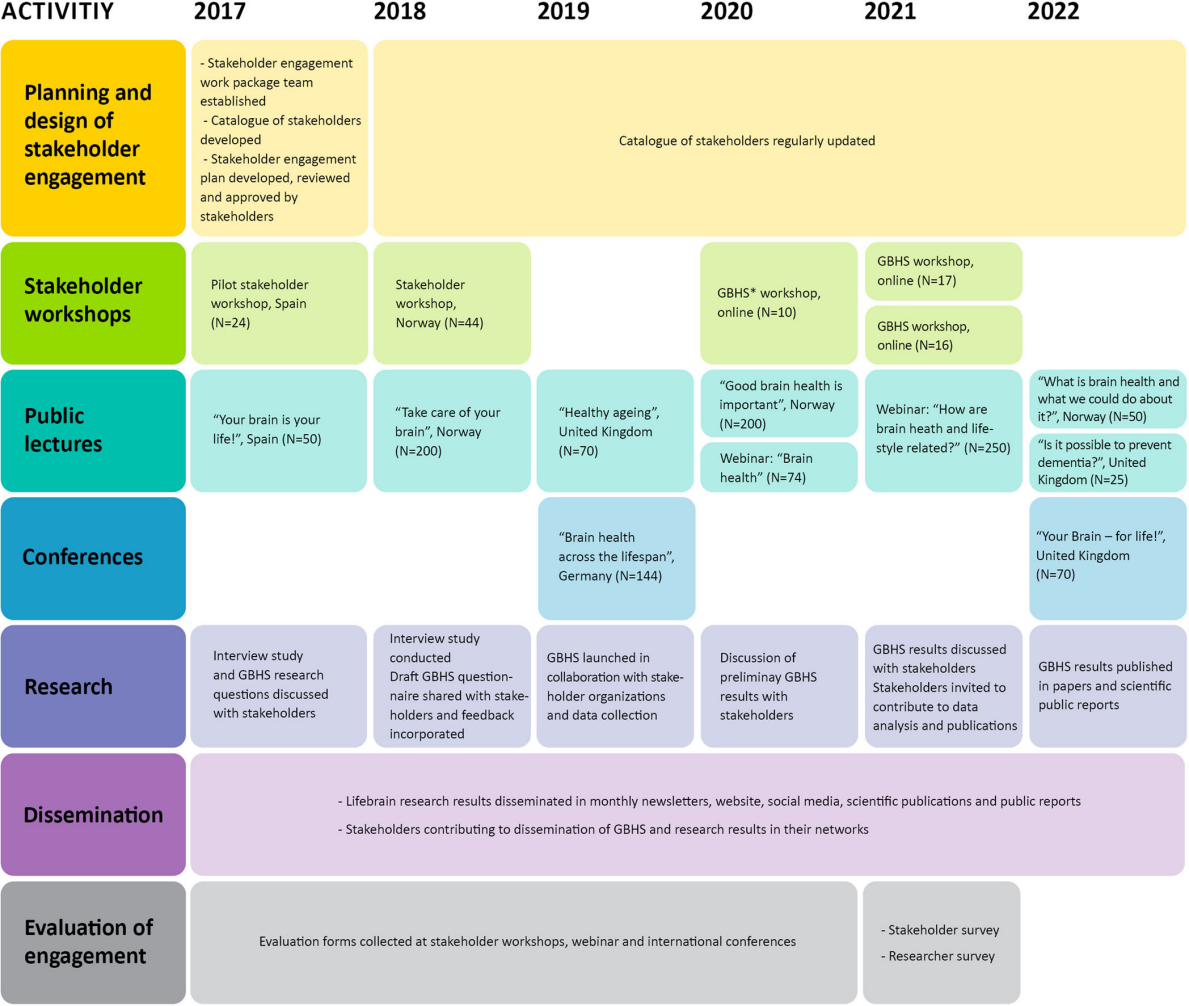
Stakeholder activities in Lifebrain along the project. GBHS, Global Brain Health Survey.

After the project started, several steps were taken to organize the stakeholder engagement process, as summarized in Figure 1. First, a stakeholder catalogue was established comprising a list of approximately 125 potential stakeholders (patient groups and organizations, cohort participants, policymakers, clinical and research centres and societies and the public) that could be relevant to engage depending on the type and location of activity planned, and the stakeholders' field of interest, level of expertise and availability. Major stakeholder organizations identified were mandated to work with brain health, such as the national brain councils and brain foundations, or focused on neurogenerative and mental disorders, such as patient organizations and research centres.

Second, a 5‐year stakeholder engagement plan was drafted outlining stakeholder engagement activities. The plan was discussed at a pilot stakeholder workshop in Barcelona in 2017, joined by Catalan and national stakeholders, such as representatives of Spanish patient‐ and interest organizations, brain health researchers, clinicians, brain research participants and policymakers, before it was finalized. The workshop participants agreed that stakeholders should be offered the opportunity to engage in activities within the framework of their competence and available resources without making stringent commitments.

Between January 2017 and June 2022, the stakeholder engagement work package organized 15 stakeholder events, including five stakeholder workshops (total number of participants *n* = 111), eight public lectures (*n* = 919) and two international conferences (*n* = 214), each held at a different location. A detailed description of the events' design and objectives is provided in Supporting Information. About half of the events were co‐organized with stakeholder organizations, with logos featured on all event materials. Stakeholders helped develop the events' programmes and promoted events in their networks and on their websites and social media platforms. No written contract was established with the organizations for their participation in the activities, and no financial compensation was provided, although Lifebrain committed to cover all costs related to the practical organization of the activities and reimburse travel and accommodation expenses of stakeholders involved in organizing physical meetings.

The stakeholder engagement work package aimed to conduct research to investigate public perspectives of brain health and invited stakeholder organizations to join such endeavour. A first study was a multisite interview study conducted in 2018.^22^ A second study consisted of an international anonymous online survey that was open between June 2019 and August 2020 (the Global Brain Health Survey, translated into 14 languages).^23^ Key stakeholders invited as survey co‐organizers helped frame survey questions, design information materials and identify relevant target groups and strategies for participant recruitment. The survey attracted 27,590 respondents across 81 countries within 14 months of availability.^24^ Stakeholders also contributed to reviewing and interpreting aggregate survey results and writing scientific papers^24^, ^25^ and public reports describing survey results.^26^, ^27^

Finally, information about the stakeholder events, the Global Brain Health Survey,^23^ and research results were disseminated with support from stakeholders.

## 2 METHODS

To evaluate our stakeholder engagement in Lifebrain, we collected feedback from consortium researchers and stakeholders. In August 2021, we sent an anonymous online survey, hereafter referred to as the ‘researcher survey’, to 40 researchers in the consortium. The survey comprised eight open‐ended questions to collect data on the researchers' experiences of stakeholder engagement, views on the benefits of and barriers to engagement, suggestions for stakeholder engagement activities and motivations for future engagement (see Supporting Information).

In April 2022, we sent an anonymous online survey, hereafter referred to as the ‘stakeholder survey’, to nine stakeholder organizations, of which seven were co‐organizers of the Global Brain Health Survey^23^ and two were research registries with whom we had collaborated closely to recruit survey respondents. The stakeholder survey comprised nine open‐ended questions regarding the stakeholders' motivations for engaging with Lifebrain, experiences of engagement, plans for following up on outcomes produced through engagement activities, ideas for activities to conduct in the remaining time of the project, suggestions for ways to improve collaboration between stakeholders and views on factors that may hinder or facilitate their engagement in future projects (see Supporting Information). Responses of the researcher and stakeholder surveys were grouped in overarching categories and summarized by the first two authors (I. B.‐L. and B. B. F.) using a content analysis approach.^28^

Additionally, and whenever practically feasible, we invited stakeholders at stakeholder events to fill in meeting evaluation forms on paper when the meetings were organized face‐to‐face or online in the case of digital events. Evaluation forms were shared at two stakeholder workshops in 2017 and 2018, one international conference in 2019, and one webinar in 2020. In total, the events gathered 286 participants, including Lifebrain researchers and stakeholders. The evaluation forms varied slightly in design and content and mainly comprised multiple‐choice questions where the respondents could either select statements to endorse or rate statements using a 5‐point Likert scale, respectively. Questions primarily focused on the relevance of topics discussed, the value of attending the event, and the stakeholder's interest in contributing to specific consortium activities. The respondents could also use free text fields to provide additional comments. The feedback from the stakeholder evaluation forms was summarized after reviewing the frequency of responses.

All data collection was done anonymously and did not require ethics clearance. Participation in the researcher and stakeholder surveys and completing evaluation forms at stakeholder events was voluntary.

## 3 RESULTS

### 3.1 Results from the researcher's survey

Eighteen Lifebrain researchers completed the survey. Most researchers reported having participated in stakeholder engagement activities, including the development and dissemination of the Global Brain Health Survey (72%), stakeholder workshops (56%), public lectures (44%) and the development of the stakeholder catalogue (44%).

Initially, the Lifebrain researchers did not have many expectations of stakeholder engagement. One respondent mentioned that ‘Lifebrain is very much a basic science endeavour’; another one explained that stakeholder engagement is ‘something most projects find difficult to implement in practice’. A few researchers hoped at the project start that stakeholder engagement would foster interest in brain research, help make the project known among other research institutions, provide insights into stakeholders' views and have a beneficial impact on researcher careers. Overall, the researchers, however, reported that their experience of stakeholder engagement in the project was positive, ‘surprisingly good’, ‘exciting and energizing’ and felt that engagement had been done ‘very professionally’. One researcher mentioned that:

The implementation of stakeholder engagement activities in Lifebrain matched the basic science nature of Lifebrain. (…) The Global Brain Health Survey has been an important and timely initiative that opened a window to engage stakeholders and policymakers more directly.

Thirteen out of 18 researchers identified the benefits of engaging stakeholders. Major benefits were related to making the research and research processes more visible, contributing to the establishment of new networks, the collection of data and the production of papers, and providing insights into stakeholder views, with the possibility to take these into account. Being specific about which contributions the stakeholders could make was seen as important:

I learned [stakeholder engagement] was useful when making specific demands; like asking for their opinion in the interviews that would be conducted with participants. If the need is very specific (…), then it is useful. If what we ask of them is unspecific or something that we expect them to do in the future, then it can come to nothing.

One respondent noted that benefits are ‘difficult to measure’ and that ‘a more intense stakeholder strategy would have allowed to better assess the impact’.

The researchers identified barriers and limitations to engaging stakeholders, including lack of time, difficulties in selecting relevant stakeholders as ‘target stakeholder groups could be potentially many’ and addressing the expectations and priorities of stakeholders and lack of common language: some researchers expressed concerns that the questions discussed required a certain scientific background. The translation of research outcomes into stakeholder advice in the form of ‘specific messages that have a clear impact’ and ‘tangible and helpful feedback to clinicians’ was also considered ‘premature’ by several researchers and a hindrance to stakeholder engagement. Some researchers found it difficult to maintain relationships with stakeholders over time as ‘stakeholders are not always institutions, but people representing these institutions, and people change’. One researcher believed that the COVID‐19 pandemic had made science outreach more difficult.

The researchers made suggestions to improve stakeholder engagement in Lifebrain, including establishing several stakeholder contacts within each project partner institution, being clear about what stakeholder engagement entails for researchers, engaging stakeholders from an early start and throughout project duration, allocating a budget for stakeholder engagement and establishing alliances with stakeholders to collect data as ‘it would be great to start data collection on a broad scale through stakeholder engagement’.

Researchers believed that several factors might motivate them to engage in future stakeholder engagement activities, such as having results that are worth communicating, having a ‘scope [that] is meaningful with respect to the aims and objectives and nature of the research project’, having sufficient funding and receiving training in stakeholder engagement and receiving ‘understandable information about what my efforts ‐ and those of other participants ‐ have contributed’. One researcher mentioned targeting specific groups as a potential motivator:

Engaging with people that are not usually easy to engage, i.e., the people that need it the most (outside of the standard highly motivated and educated people that always find their way into research, and already are able to find the information and help they need) or talking to people that have practice‐based experience with these groups

### 3.2 Results from the stakeholder survey

Seven out of nine stakeholder organizations completed the survey. Most had agreed to collaborate with us on the Global Brain Health Survey^23^ because they found it to be within their field of interest and believed that it had ‘theoretical and practical implications for wellbeing and quality of life’ and was ‘an opportunity to involve volunteers in a multinational study’. The stakeholders thought they could use survey results in future information and lobbying campaigns related to brain health and to better understand the views of their target audience regarding brain health.

Most stakeholders experienced that the collaboration with the Lifebrain researchers was good, ‘easy and successful’, and appreciated being invited to follow‐up workshops about survey results. They expressed interest in attending similar activities with Lifebrain, such as public meetings to disseminate results from the Global Brain Health Survey,^23^ participating in the writing of scientific publications on survey results, and conducting research to explore the impact of the COVID‐19 pandemic on attitudes to research. They recommended involving stakeholders in future projects like Lifebrain to organize public events and conferences, help conduct the research, raise awareness about the project and increase the number of participants. Factors they believed could hinder their participation in similar projects in the future included lack of budget, limited resources in their organization, time constraints, insufficient communication between researchers and stakeholders or conducting projects on themes that are irrelevant to them. As mentioned by one stakeholder:

Connection of the themes of the project with our strategy [is an important factor]: for instance, brain health is an important topic at this moment, but it could be that we focus more on specific brain diseases in the future

The researchers mentioned factors that could facilitate their participation in future projects, including allocated financial resources, collaborating with ‘enthusiastic researchers willing to communicate’, and the conduct of ‘activities that are beneficial for all parties, fit very well with our strategy and do not require big time investment’.

### 3.3 Results from the evaluation forms shared at stakeholder events

In total, 104 evaluation forms were collected. Overall, the stakeholders found the stakeholder events interesting, informative, useful, and of appropriate duration. They believed participating in the events helped them gain a good overview of challenges and opportunities in the field of brain health and were willing to participate in future Lifebrain activities.

## 4 DISCUSSION

For 5.5 years, the Lifebrain consortium engaged with various stakeholders through the organization of stakeholder events and by researching public perceptions of brain health. Evaluation of our stakeholder engagement shows that stakeholders and researchers experienced activities as meaningful. Although the researchers initially considered the basic science nature of Lifebrain as one potential hindrance to stakeholder engagement, their commitment to engage with stakeholders grew as they experienced the benefits of engagement, including the development of a strong collaborative network, as well as survey data acquisition and the publication of scientific papers and public reports. The stakeholders engaged with the consortium because they found activities relevant to their field of interest and aimed to exploit research results in their work. Although many engagement activities were conducted, stakeholder engagement was constrained by time pressure, challenges in communicating complex scientific issues and limited time and resources to maintain relationships with stakeholders over time.

The benefits and challenges of stakeholder engagement experienced in Lifebrain are in line with results from recent systematic reviews,^6^, ^29^ reporting that engaging stakeholders in health research projects helped enhance the quality and appropriateness of research and relevance of research questions, identify appropriate recruitment strategies, increase enrolment rates and interpret results. Thanks to the input and support from stakeholders, the Global Brain Health Survey^23^ gathered a very large number of respondents to a brain health survey, probably because the stakeholders contributed to making the survey attractive and accessible to the general public. In line with findings by Concannon et al.,^2^ stakeholder engagement in Lifebrain enhanced mutual learning and understanding by stakeholders and researchers: the stakeholders learned about brain health research, and the researchers gained new insights into what motivates stakeholders to promote brain health and value research. As in other projects, we early on realized that stakeholder engagement required significant effort and that we had to limit our ambitions due to constraints on time and human resources.^1^, ^2^ Thus, we collaborated closely with only a restricted number of stakeholders, mostly umbrella patient organizations, as these had resources to engage with us and could make real contributions.^8^

Engaging stakeholders in basic brain research is challenging as study findings often have limited direct practical implications.^13^ In the stakeholder engagement work package, the research on public perceptions of brain health complemented our basic research efforts and provided additional knowledge of translational value and practical relevance to healthcare and public health policies. Inviting stakeholders to collaborate on investigating public perceptions of brain health, providing them with visibility as co‐organizers of the research, and enabling them to use findings to inform their work, was a strong motivator for engagement. There are usually few incentives encouraging researchers to engage with stakeholders.^20^ Developing the Global Brain Health Survey^23^ enabled researchers in the consortium to engage with audiences and professionals they typically would not interact with directly while collecting research data. Future projects should consider conducting research that bridges basic science and the stakeholders' mandate to address societal health challenges.

Recommendations have been made to establish clear ground rules for engagement activities and inform stakeholders about these rules.^1^, ^30^ This may include delineating stakeholder roles, responsibilities and expectations, establishing well‐articulated plans for engagement activities and decision‐making processes,^30^ determining levels of engagement for each stakeholder group, outlining strategies for resolving conflicts and power imbalance issues and informing stakeholders about how their input will be integrated.^31^, ^32^ At the project start, we developed a stakeholder engagement plan, although it primarily outlined activities we aimed to conduct and did not articulate stringent rules for engagement. For each planned activity, we approached the relevant local stakeholders, discussed mutual expectations and made agreements for their contributions. This provided the stakeholders with sufficient flexibility to engage depending on their availability, interest, competence, resources and level of ambition and helped create an environment of trust and reciprocal respect.^6^ Such flexibility was useful when the COVID‐19 pandemic led to lockdowns and stringent restrictions on the organization of face‐to‐face meetings. Although we had to reduce on‐site activities, we managed to pursue contact with stakeholders digitally and organized stakeholder events online, thus limiting the impact of the pandemic on our activities.

Overall, we did not encounter conflicts in our interactions with stakeholders. However, researchers raised concerns that results from the Global Brain Health Survey^23^ may be rendered public when sharing aggregate results with stakeholders before publication in scientific journals, thus bypassing regular peer‐review and quality assurance processes. Such concerns were not due to a lack of trust of the co‐organizers but rather grounded in the thoughts that nonscientific stakeholders may not be familiar with rules and practices for scientific publication and may have an interest in acting immediately on new knowledge or by selecting specific outcomes. In the absence of detailed written agreements regarding their role and responsibilities in the research, the stakeholders found it to be at times unclear which survey data would be accessible for their use, and some stakeholders, not being scientists themselves, were uncomfortable in participating in the writing of scientific papers. In future projects, research activities are planned, it would be important already to clarify the respective responsibilities and contributions of stakeholders and researchers in the early stages of the project.

Finding good tools to evaluate the full impact of stakeholder engagement is difficult. Engagement activities are often interconnected, linked to several stages of the research process, and difficult to measure.^4^ This may cause scepticism or indifference to the potential value of stakeholder engagement. In Lifebrain, we used surveys and evaluation forms to assess our stakeholder engagement. This gave some insight into the perceived value of interactions between researchers and stakeholders, although it cannot be seen as a complete assessment of our stakeholder engagement.^33^ Frameworks^34^ and guidelines for stakeholder engagement exist, emphasizing the importance of predefined and validated evaluation tools, but such tools are not tailored to stakeholder engagement conducted in basic science. Future research is needed to develop specific tools to evaluate engagement activities. When planning future projects, researchers may use such tools early on, a priori, as an embedded component of the research process to support more intentional planning, development and reporting of stakeholder engagement activities.

## 5 CONCLUSION

It is particularly challenging for brain researchers in basic science to ensure academic excellence and societal impact simultaneously. Our experience from the Lifebrain project illustrates that stakeholder engagement is possible and can have a beneficial impact. The Norwegian Health Directorate recently published a report summarizing the experiences of 3 years with a national brain health strategy and outlined future steps.^35^ The report relied on results from the Global Brain Health Survey^23^ as a basis for outlining future work to increase brain health awareness. Achieving such an impact on health policy would not have been possible without the support and engagement of stakeholders. Engagement activities are more likely to be successful if they benefit both researchers and stakeholders.

## AUTHOR CONTRIBUTIONS

Isabelle Budin‐Ljøsne, Barbara B. Friedman, William F. C. Baaré, David Bartrés‐Faz, Rebecca B. Carver, Christian A. Drevon, Klaus P. Ebmeier, Anders M. Fjell, Paolo Ghisletta, Rogier Kievit, Richard N. Henson, Kathrine S. Madsen, Laura Nawijn, Sana Suri, Cristina Solé‐Padullés, Kristine B. Walhovd and Enikő Zsoldos were involved in the design and data collection of the study. Isabelle Budin‐Ljøsne and Barbara B. Friedman conducted the stakeholder engagement evaluation process and analysed data. William F. C. Baaré, David Bartrés‐Faz, Christian A. Drevon, Klaus P. Ebmeier, Sana Suri and Cristina Solé‐Padullés made a substantial contribution to the analysis of data. Isabelle Budin‐Ljøsne and Barbara B. Friedman drafted the manuscript. Isabelle Budin‐Ljøsne, Barbara B. Friedman, William F. C. Baaré, David Bartrés‐Faz, Rebecca B. Carver, Christian A. Drevon, Klaus P. Ebmeier, Anders M. Fjell, Paolo Ghisletta, Rogier Kievit, Richard N. Henson, Kathrine S. Madsen, Laura Nawijn, Sana Suri, Cristina Solé‐Padullés, Kristine B. Walhovd and Enikő Zsoldos substantively revised the manuscript and approved the submitted version. Isabelle Budin‐Ljøsne is the guarantor for the article.

## CONFLICT OF INTEREST STATEMENT

The authors declare no conflict of interest.

## ETHICS STATEMENT

All necessary ethics approvals for the study have been provided for, including approvals from the Regional Ethics Committee of Norway and the Norwegian Centre for Research Data.

## Supporting information

Supplementary information.

## Data Availability

The data that support the findings of this study are available from the corresponding author upon reasonable request.
