# Supplementary material for: Stakeholder engagement in European brain research: Experiences of the Lifebrain consortium
Source: Health Expect. 2023 Mar 29;26(3):1318–26. doi: 10.1111/hex.13747 (PMC10154816; doi:10.1111/hex.13747)
Supplement: Supplementary file 1 — Supplementary information. [file HEX-26--s001.docx]

**Supplementary material - Stakeholder engagement in European brain research: Experiences of the Lifebrain consortium**

1. **Overview of stakeholder events in Lifebrain**
2. **Questionnaire – Researcher survey**
3. **Questionnaire – Stakeholder survey**

**1) Overview of stakeholder events in Lifebrain**

| **Stakeholder events /co-organizers** | **Stakeholders (N=number)** | **Objectives / Design** |
| --- | --- | --- |
| ***Stakeholder workshops*** |  |  |
| Pilot stakeholder workshop (Barcelona, Spain, 2017) | N=24 Catalan and national representatives of patient and interest organizations, brain health researchers and clinicians, research participants, policymakers | - Introduce Lifebrain to stakeholders  - Discuss draft stakeholder engagement plan  - Discuss role of stakeholders in the project and preferred forms of collaboration  - Sketch of research questions discussed with stakeholders |
| Stakeholder workshop (Oslo, Norway, 2018)  Co-organizer: Norwegian Brain Council | N=44 Brain health researchers, representatives from medical and patient associations, policymakers | - Discuss avenues for the promotion of brain health across the lifespan |
| Global brain health survey workshop (online, 2020) | N=10 representatives of survey co-organizing organizations | - Discuss preliminary survey results pertaining to brain health perceptions  - Outline strategies to inform policymakers about survey outcomes |
| Global brain health survey workshop (online, spring 2021) | N=17 Survey co-organizing organizations, research registries, and researchers conducting similar surveys | - Discuss key messages to convey to policymakers based on survey results  - Develop plans for results publication and dissemination  - Agree on stakeholder contributions in publications  - Share experiences from similar surveys and national spin-offs of the Global Brain Health Survey |
| Global brain health survey workshop (online, fall 2021) | N=16 Survey co-organizers, research registries and researchers conducting similar surveys | - Discuss results from draft report presenting survey results pertaining to respondents’ motivations to take care of their brain health  - Outline strategies for report dissemination |
| ***Public lectures/webinars*** |  |  |
| “Your brain is your life” (Barcelona, Spain, 2017) | N=50 lay people, researchers and patient organization representatives | - Presentation of Lifebrain research in English and Spanish) |
| “Take care of your brain!” (Oslo, Norway, 2018)  Co-organizer: Norwegian Brain Council | N=200 lay people, researchers and patient organization representatives | - Presentation of Lifebrain research  - Representative from Norwegian Brain Council moderating the event |
| “Healthy ageing” (Cambridge, United Kingdom, 2019)  Co-organizer: University of Cambridge | N=70 lay people, researchers, patient organization representatives and research participants | - Presentation of Lifebrain research and brain research by external invited speaker  - Draft survey questionnaire shared with audience for feedback on design and content |
| “Good brain health is important!” (Oslo, Norway, 2019)  Co-organizer: Norwegian Brain Council | N=200 lay people, researchers, patient organization representatives | - Presentation of Lifebrain research  - Official launch of the Global Brain Health Survey  - Representative from Norwegian Brain Council moderating the event |
| Webinar on brain health (online, 2020)  Co-organizer: Norwegian Brain Council | N=74 researchers, health care professionals, patient organization representatives and lay people | - Presentation of Lifebrain research  - Discussion of research implications for policymaking |

| “How are brain health and lifestyle related?” (Norway/online, 2021)  Co-organizer: Norwegian Brain Council | N=250 lay people and patient organization representatives | - Presentation of survey results  - Panel session with Lifebrain/external researchers and policymakers to discuss results  - Representative from Norwegian Brain Council moderating the event |
| --- | --- | --- |
| “What is brain health and what we could do about it?” (Lom, Norway, 2022). Co-organizer: Municipality of Lom | N=50 lay people | - Presentation of survey results  - Panel session with local people on brain health |
| “Is it possible to prevent dementia?” (Oxford, United Kingdom, 2022). Co-organizer: James Str. Tavern | N=25 lay people, researchers, healthcare professionals | - Presentation of Lifebrain results  - Discussion on brain health |
| ***Conferences*** |  |  |
| “Brain Health across the lifespan” (Berlin, Germany, 2019)  Co-organizers: Silver Santé Study, German Brain Council | N=144 researchers, healthcare professionals, patient organization representatives | - Presentation of Lifebrain and Silver Santé research  - Discussion of research implications by representative of German brain |
| “Your brain for life – Closing conference for the Lifebrain consortium (Oxford, United Kingdom, 2022) | N=70 researchers | - Presentation of Lifebrain research findings  Discussion of stakeholder engagement experiences of Lifebrain |

**2) Questionnaire – Researcher survey**

1. Which Lifebrain stakeholder engagement activities have you participated in? Select any from the following list:

- Setting up a list of stakeholders
- Planning and implementation of the interview study (See paper in The Gerontologist)
- Development and dissemination of the Global Brain Health Survey
- Stakeholder workshops
- Public lectures/webinars
- Conference in Berlin
- Other stakeholder engagement activities (for e.g., local Science Festivals)
- None

2. What was your initial expectation of stakeholder engagement in Lifebrain, if any?

3. What is your actual experience with stakeholder engagement in Lifebrain? What do you think about stakeholder engagement in Lifebrain?

4. In your opinion, are there any benefits of engaging stakeholders in Lifebrain?

5. In your opinion, what are the barriers/limitations of engaging stakeholders in Lifebrain?

6. Do you have ideas/suggestions for improving stakeholder engagement in Lifebrain?

7. Do you have any new ideas for specific stakeholder engagement activities in Lifebrain?

8. What would motivate you to engage in future stakeholder engagement activities in other research projects?

**3) Questionnaire – Stakeholder survey**

1. Why did your organization agree to be an official co-organizer of the Global Brain Health Survey?

2. How will the results of the survey be useful for your organization?

3. What is your experience with participating as a survey co-organiser in Lifebrain?

4. What activities would you like to conduct with Lifebrain in the remaining timeframe of the project (the project will end in June 2022)?

5. Do you have suggestions for ways to improve collaboration between your organization and research projects like Lifebrain?

6. What types of activities would you like to conduct in the future with research projects like Lifebrain?

7. In your opinion, which factors could hinder your participation in similar projects in the future?

8. In your opinion, which factors could facilitate your participation in similar projects in the future?

9. Any further comments/ suggestions/ experiences you would like to share with us?
